# QuickStats

**Published:** 2015-05-29

**Authors:** FROM THE NATIONAL CENTER FOR HEALTH STATISTICS

**Figure:**
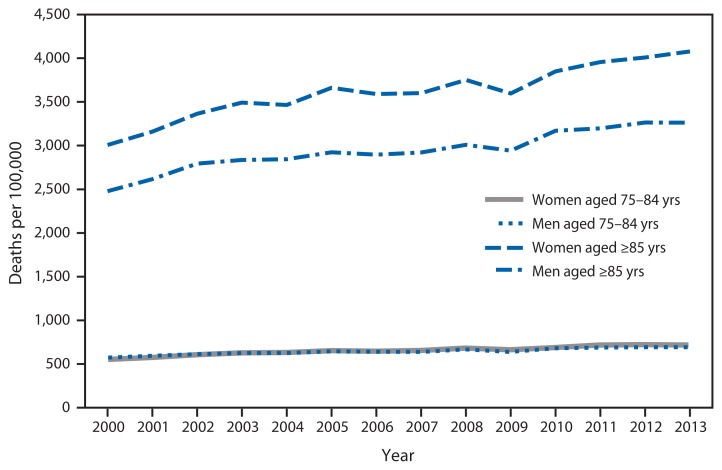
Death Rates* from Dementia^†^ Among Persons Aged ≥75 Years, by Sex and Age Group — United States, 2000–2013 * Per 100,000 population. ^†^ Deaths from dementia include underlying and contributing causes of death coded F01 (vascular dementia), F03 (unspecified dementia) or G30 (Alzheimer’s disease) according to the *International Classification of Diseases, 10th Revision*.

During 2000–2013, death rates for dementia per 100,000 population increased for both men and women among persons aged 75–84 years and ≥85 years. Among persons aged 75–84 years, the rate increased 21% for men and 31% for women. Among persons aged ≥85 years, the rate increased 32% for men and 36% for women. Among persons aged ≥85 years, death rates were higher for women than men throughout the period, with death rates 25% higher among women than men in 2013 (4,077.4 versus 3,261.6 per 100,000 population).

**Source:** National Vital Statistics System. Multiple cause of death data, 2000–2013. Available at http://wonder.cdc.gov/mcd-icd10.html.

**Reported by:** Ellen A. Kramarow, PhD, ekramarow@cdc.gov, 301-458-4325.

